# Systemic Therapy De-Escalation in Early-Stage Triple-Negative Breast Cancer: Dawn of a New Era?

**DOI:** 10.3390/cancers14081856

**Published:** 2022-04-07

**Authors:** Ravi Kumar Gupta, Arya Mariam Roy, Ashish Gupta, Kazuaki Takabe, Ajay Dhakal, Mateusz Opyrchal, Pawel Kalinski, Shipra Gandhi

**Affiliations:** 1 Department of Internal Medicine, Larkin Community Hospital, South Miami, FL 33143, USA; rgupta@larkinhospital.com; 2 Department of Medicine, Roswell Park Comprehensive Cancer Center, Buffalo, NY 14263, USA; arya.roy@roswellpark.org (A.M.R.); ashish.gupta@roswellpark.org (A.G.); pawel.kalinski@roswellpark.org (P.K.); 3 Department of Immunology and Surgical Oncology, Roswell Park Comprehensive Cancer Center, Buffalo, NY 14263, USA; kazuaki.takabe@roswellpark.org; 4 Department of Gastroenterological Surgery, Yokohama City University Graduate School of Medicine, Yokohama 236-0004, Japan; 5 Department of Surgery, Niigata University Graduate School of Medicine and Dental Sciences, Niigata 951-8510, Japan; 6 Department of Breast Surgery and Oncology, Tokyo Medical University, Tokyo 160-8402, Japan; 7 Department of Surgery, Jacobs School of Medicine and Biomedical Sciences, State University of New York, Buffalo, NY 14263, USA; 8 Department of Medicine, University of Rochester Medical Center, Rochester, NY 14648, USA; ajay_dhakal@urmc.rochester.edu; 9 Department of Medicine, Indiana University Simons Comprehensive Cancer Center, Indianapolis, IN 46202, USA; mopyrch@iu.edu; 10 Department of Immunology, Roswell Park Comprehensive Cancer Center, Buffalo, NY 14263, USA

**Keywords:** triple-negative breast cancer, de-escalation, targeted therapy, BRCA mutations, chemotherapy, neo adjuvant treatment, tumor infiltrating lymphocytes, biomarkers, immunotherapy

## Abstract

**Simple Summary:**

Triple-negative breast cancer is a life-threatening disease, even when identified at early stages. Recent advances have allowed the improvement of life expectancy via a personalized approach with the addition of newer chemotherapies, immunotherapies, and targeted therapies, but at the cost of added side effects. It has become increasingly clear that not all patients need such aggressive treatment. Here, we provide an overview of emerging opportunities to use less toxic therapies in patients at lower risk of recurrence or with mutations that can be effectively targeted using novel approaches. We provide a comprehensive review of completed and ongoing clinical trials with information on how to best stratify these patients for treatments to obtain maximum benefit without unnecessary toxicities.

**Abstract:**

Early-stage triple negative breast cancer (TNBC) has been traditionally treated with surgery, radiation, and chemotherapy. The current standard of care systemic treatment of early-stage II and III TNBC involves the use of anthracycline-cyclophosphamide and carboplatin-paclitaxel with pembrolizumab in the neoadjuvant setting followed by adjuvant pembrolizumab per KEYNOTE-522. It is increasingly clear that not all patients with early-stage TNBC need this intensive treatment, thus paving the way for exploring opportunities for regimen de-escalation in selected subgroups. For T1a tumors (≤5 mm), chemotherapy is not used, and for tumors 6–10 mm (T1b) in size with negative lymph nodes, retrospective studies have failed to show a significant benefit with chemotherapy. In low-risk patients, anthracycline-free chemotherapy may be as effective as conventional therapy, as shown in some studies where replacing anthracyclines with carboplatin has shown non-inferior results for pathological complete response (pCR), which may form the backbone of future combination therapies. Recent advances in our understanding of TNBC heterogeneity, mutations, and surrogate markers of response such as pCR have enabled the development of multiple treatment options in the (neo)adjuvant setting in order to de-escalate treatment. These de-escalation studies based on tumor mutational status, such as using Poly ADP-ribose polymerase inhibitors (PARPi) in patients with BRCA mutations, and new immunotherapies such as PD1 blockade, have shown a promising impact on pCR. In addition, the investigational use of (bio)markers, such as high levels of tumor-infiltrating lymphocytes (TILs), low levels of tumor-associated macrophages (TAMs), and complete remission on imaging, also look promising. In this review, we cover the current standard of care systemic treatment of early TNBC and review the opportunities for treatment de-escalation based on clinical risk factors, biomarkers, mutational status, and molecular subtype.

## 1. Introduction

Triple-negative breast cancer (TNBC) is a subtype of breast cancer that lacks estrogen receptor (ER), progesterone receptor (PR), and human epidermal growth factor receptor 2 (HER2) expression. Approximately 10–15% of breast cancers are triple-negative. These are typically more aggressive and associated with a higher rate of relapse. In addition, they have a higher predisposition to involve visceral organs like the lungs, liver, and brain, leading to a significantly shorter survival than other subtypes [1]. Thus, there have been significant ongoing efforts to develop optimal treatment strategies to treat both early and advanced TNBC. 

In the present era of molecular subtyping, great progress has been made in finding effective targeted therapies for most subtypes of breast cancer. As TNBC lacks receptor expression, such as ER/PR, the vast array of endocrine therapy agents, such as selective estrogen receptor modulators (SERMS) and aromatase inhibitors, are out of treatment focus. Therefore, the standard systemic treatment option for early-stage TNBC was, until recently, limited to (neo)adjuvant chemotherapy followed by surgery and radiation [2]. The approval of pembrolizumab (July 2021) in combination with carboplatin/paclitaxel and doxorubicin/cyclophosphamide in early TNBC in the neoadjuvant setting in KEYNOTE-522, with continuation of pembrolizumab in the adjuvant setting, shifts the standard of care regimen for early-stage TNBC towards an even more intensive chemotherapy backbone, now with an immune checkpoint inhibitor (ICI). However, immune related adverse events (irAEs) were observed in 43.6% of patients with this combination vs. 21.9% with chemotherapy alone. Some of these irAEs could be fatal and life-threatening [2] and, hence, this begs the question of whether patients need ICI or if it is possible to de-escalate treatment for select individuals. 

De-escalation has been successfully accomplished in the surgical field for breast cancer. From Halsted’s radical mastectomy described over 100 years ago with axillary lymph node dissection (ALND), which were profoundly morbid procedures, to the current standard-of-care of breast conservation therapy (BCT) with lumpectomy and/or sentinel lymph node biopsy (SLNB) [3], the field has made significant progress. Multiple trials have demonstrated that breast conservation therapy (BCT) i.e., lumpectomy with radiation, is at least equivalent to mastectomy alone in terms of survival outcomes [4]. In addition, neoadjuvant chemotherapy (NACT) has enabled more and more women to receive BCT [5]. Over the last five years, both an improved understanding of the subtypes of TNBC as well as identification of targeted therapies for mutations have contributed to a movement towards de-escalation of systemic therapy [6]. De-escalating therapy has multiple potential benefits, including reduced toxicity, improved quality of life, improved cost-effectiveness, and better compliance with therapy, while maintaining good clinical outcomes. 

De-escalating systemic therapy has been attempted in several ways—to administer less toxic/less aggressive regimens in a (neo)adjuvant setting, to stratify patients by identifying low clinical or molecular risk subgroups in early TNBC to avoid aggressive regimens, or to decrease the duration of therapies [7]. In addition, several targeted therapies are under investigation for use in early TNBC, including anti-angiogenic agents, androgen receptor blockers, and epidermal growth factor receptor (EGFR) targeted agents; however, their use is currently limited to clinical trials [8].

In this review, we focus on the de-escalation of systemic therapy in early TNBC and review the current literature in this field as well as ongoing and completed trials.

## 2. Current Standard of Care of TNBC

The choice of treatment in early TNBC largely depends on the primary tumor size, number of lesions, and lymph node (LN) involvement. Current national and international guidelines recommend neoadjuvant or adjuvant chemotherapy for early TNBC with tumor size ≥ 1 cm and/or with LN involvement, especially with ≥1 ipsilateral LN with metastases > 2 mm. But when it comes to stage 1 TNBC, especially pT1N0M0, there are no clear data since most of these patients were excluded from definitive clinical trials [9].

There is discordance among major guidelines: National Comprehensive Cancer Network (NCCN), American Society of Clinical Oncology guidelines (ASCO), European Society for Medical Oncology (ESMO), St. Gallen International Expert Consensus, Dutch guidelines regarding the best clinical practice for early TNBC, which is described in Table 1 [10,11,12,13,14,15]. Most of the guidelines recommend against adjuvant chemotherapy for T1aN0 TNBC and recommend systemic chemotherapy (neo/adjuvant) for T1cN0 TNBC. ESMO guidelines recommend adjuvant chemotherapy for pT1 tumors, with the possible exclusion of low-risk special histological subtypes and very early (T1aN0) tumors [12]. There is no consensus regarding systemic chemotherapy among these guidelines for T1bN0 TNBC, which makes decision making strenuous for clinicians and patients [10,11,12,13,14,15].

The mainstay for early-stage TNBC with tumor greater than 2 cm in size (T2 or more) (prior to KEYNOTE-522 data) was neoadjuvant chemotherapy (NACT) followed by definitive surgery with or without adjuvant treatment (if residual disease). NACT was also used for T1 tumors if upfront surgery would provide an inferior cosmetic outcome and downstaging was essential. NACT also provides additional benefit in patients with locally advanced breast cancer who are not candidates for breast-conserving surgery (BCS) and wish for breast conservation, or those who are unlikely to have a good cosmetic outcome with upfront BCS [10].

The most common chemotherapy regimen in the United States for use in a (neo) adjuvant setting for cT1N0 is anthracycline and cyclophosphamide (AC) given in dose-dense schedule followed by weekly paclitaxel or dose-dense paclitaxel for a total of 4–5 months [11]. The rationale for using this regimen came from the meta-analysis carried out by the Early Breast Cancer Trialists’ Collaborative Group (EBCTCG) in 2012, which showed that anthracycline-based regimens had similar or even better outcomes over historical cyclophosphamide, methotrexate, and fluorouracil (CMF) regimen. In addition, adding taxanes was associated with a reduction in recurrence rate and breast cancer-specific mortality. This led anthracycline and taxane (AT)-based regimens to become the standard treatment for operable breast cancer [16]. 

Although AC followed by taxane is superior for high-risk patients, alternative therapies are considered for patients with early TNBC who cannot receive anthracyclines, for example, those with a history of cardiac disease or extensive cardiac comorbidities and those who are unwilling to accept the risks of anthracycline-based therapies. For such patients, taxane-based treatment is offered; the common regimen used is docetaxel and cyclophosphamide (TC) [17]. CMF is another alternative regimen that can be used for those who have extensive peripheral neuropathy or cardiac comorbidities preventing the use of taxanes and anthracyclines [16]. 

For patients who cannot complete the full treatment course of NACT, the remainder of the planned course of chemo is usually completed in the adjuvant setting after careful consideration of adjustments for toxicities. For patients who have completed the standard NACT, and if they have attained complete pathologic response (pCR), then further adjuvant chemotherapy is not required. However, if the patients did not achieve complete pCR after NACT, they have a higher risk of disease recurrence. For those patients, adjuvant capecitabine was recommended after the promising results of the CREATE-X trial. It showed that patients with HER2 negative breast cancer with residual disease after NACT who received adjuvant capecitabine had higher rates of five-year disease-free survival (DFS) and overall survival (OS) when compared to those who received no further treatment. Subgroup analysis suggested that the improvement in DFS was mainly due to the improvement in outcomes among patients with early TNBC with 5-year DFS of 69.8% with capecitabine vs. 56.1% in the control group (HR 0.58, 95% CI, 0.38 to 0.87) and 5-year OS of 78.8% vs. 70.3% in the capecitabine vs. the control group (HR 0.52, 95% CI, 0.30 to 0.90) [18]. Recently, a PARPi, olaparib, was approved for patients with Her 2 negative breast cancer with germline BRCA mutation who have residual disease after NACT, based on the positive findings from the OlympiA trial that showed 3 year-invasive DFS of 85.9% in olaparib vs. 77.1% in the control group (HR 0.58, 99.5% CI, 0.41 to 0.82) and 3-year distant DFS of 87.5% in olaparib vs. 80.4% in the control group (HR 0.57, 99.5% CI, 0.39 to 0.83) [19]. According to recently published data, olaparib also significantly improved the OS. The 3-year OS rate in the olaparib arm vs. placebo was 92% vs. 89.1% (HR 0.68; 98.5% CI, 0.47 to 0.97, *p* = 0.0009) [20]. This highlights the need for better personalized strategies in the neoadjuvant setting to improve pCR, so that additional adjuvant treatment can be avoided. 

In July 2021, pembrolizumab was approved by the US Food and Drug Administration (FDA) for combined use with chemotherapy in high-risk TNBC in the neoadjuvant and adjuvant setting based on the KEYNOTE-522 trial. In this phase III trial, patients with previously untreated stage II or III TNBC were randomly assigned to receive NACT with or without pembrolizumab every three weeks during the NACT and continued for another nine cycles after surgery, regardless of the pathologic response to the neoadjuvant treatment. The trial used a regimen of four cycles of paclitaxel and carboplatin plus pembrolizumab followed by AC plus pembrolizumab as neoadjuvant treatment and then adjuvant pembrolizumab for a total of nine cycles. The trial showed that the pCR rates were higher among those who received pembrolizumab plus chemotherapy than chemotherapy alone (64.8% vs.51.2%). This improvement in pCR rates was seen in both programmed cell death (PD-L1) positive and negative stage II or III TNBC. The percentage of patients alive at 18 months without disease progression/local or distant recurrence in the pembrolizumab + chemotherapy group was 91.3% and, in the chemotherapy alone group, was 85.3% (HR for disease progression 0.63, 95% CI 0.43 to 0.93) [2]. Follow-up data presented at the ESMO conference showed that the addition of pembrolizumab showed improvement in the 36-month event-free survival (EFS), 84% in the pembrolizumab group vs. 77% in the placebo group, with 37% reduction in events (HR 0.63, 95% confidence interval 0.48 to 0.82). EFS improvement was also independent of the PD-L1 status [21]. It was also found that the addition of pembrolizumab was more beneficial in patients who had residual disease when compared to those who attained pCR. In those who did not attain pCR, the 3-year EFS was 67.4% in the pembrolizumab + chemo group and 56.8% in the chemotherapy alone group. Meanwhile, in the patients who attained pCR, the 3-year EFS was 94.4% in the pembrolizumab + chemotherapy group and 92.5% in the chemotherapy alone group [21,22]. Thus, the current standard of care for tumor stage T1c, nodal stage N1-2, or tumor stage T2-4, nodal stage N0-2 is neoadjuvant pembrolizumab with chemotherapy.

Similar findings were observed in the IMPassion031 clinical trial. IMPassion031 is a phase III randomized clinical trial for patients with TNBC with tumor size > 2 cm (*N* = 333). Patients were randomized to receive nab-paclitaxel followed by doxorubicin and cyclophosphamide, with or without atezolizumab, followed by surgery. After surgery, 11 doses of atezolizumab were administered every 3 weeks in the immunotherapy group. pCR was significantly improved in 57.6% of patients in the atezolizumab plus chemotherapy group, and in approximately 41% of the patients in the placebo plus chemotherapy group. In the PD-L1-positive population, pCR was observed in 68.8% of the patients in the atezolizumab group vs. 49.3% in the placebo group [23]. 

The GeparNUEVO trial also investigated the effects of an anti-PD-L1 checkpoint inhibitor in the neoadjuvant setting. This trial investigated the effect of the addition of neoadjuvant durvalumab to anthracycline/taxane-based chemotherapy. In this trial, they randomized cT1b-cT4a-d TNBC patients to receive either durvalumab or placebo along with neoadjuvant nab-paclitaxel followed by epirubicin plus cyclophosphamide. The pCR rates were higher in the durvalumab group compared to the placebo group, but the difference was not statistically significant. However, durvalumab added to neoadjuvant chemotherapy has shown a statistically significant improvement in long-term outcomes. The 3-year distant disease-free survival (DDFS) and OS were 91.4% and 95.1% in the durvalumab group, compared to 79.5% and 83.1% in the placebo group, respectively, which was statistically significant [24]. 

As discussed, with the approval of checkpoint inhibitors along with chemotherapy in the neoadjuvant setting, the treatment for early-stage TNBC has become very aggressive, associated with several irAEs. They key question now is whether all patients need this aggressive treatment, or can the treatment be de-escalated for patients selected based on tissue biomarkers or tumor/genetic mutations.

## 3. Molecular Heterogeneity of TNBC

In this section, we discuss the various subtypes of TNBC, which form the basis of current and future efforts of de-escalation. TNBC has immense heterogeneity (transcriptomic, genomic, and histopathological), which may account for the varied response to systemic therapy. A pivotal study by Lehman et al. analyzed 587 TNBC patients and subdivided TNBCs according to gene expression clustering into six subtypes [25]: basal-like 1 (BL1), basal-like 2 (BL2), mesenchymal (M), mesenchymal-stem like (MSL), immunomodulatory (IM), and luminal androgen receptor type (LAR). Each subtype is associated with specific genetic alterations. These subtypes were found to significantly differ in response to NACT; pCR rates were 41% for BL1, 18% for BL2, and 29% for LAR [26].

The evolution of our understanding of this heterogeneity at the molecular level along with the development of novel drugs has helped facilitate more personalized anticancer treatment for patients with TNBC, which has led to increased treatment options and a more complicated decision process. The BL1 subtype has abnormal expression of cell-cycle regulating and DNA repair-related genes with sensitivity to PARPi and cisplatin [27]. There is abnormal activation of signaling pathways such as EGFR, MET, NGF, Wnt/beta-catenin, and IGF-1R, and, therefore, potential targeted options include mTOR inhibitors and growth factor inhibitors (lapatinib, gefitinib, and cetuximab) [27]. The M subtype has sarcoma-like or squamous-epithelial-like features and is relatively chemo-resistant, but it is sensitive to mTOR inhibitors or drugs targeting epithelial-to-mesenchymal transition [28]. The MSL subtype expresses a low level of cell-proliferation-related genes and high levels of stemness-related genes; HOX genes; and mesenchymal stem-cell specific markers that may be treated with PI3K inhibitors, Src antagonists, or antiangiogenic drugs [27]. The IM subtype is significantly enriched in immune-cell associated genes and pathways, such as Th1/Th2, NK cell and B-cell receptor signaling pathway, dendritic cell (DC) pathway, interleukin (IL)-7, IL-12, and T-cell receptor pathway [29]. This subtype may be sensitive to checkpoint inhibitors targeting PD1, PD-L1, and CTLA-4 [27]. The LAR subtype represents 10–15% of TNBCs, is relatively chemotherapy-resistant, has a low rate of pCR, and is characterized by >10% expression of the androgen receptor [30]. Preclinical models have shown that the LAR subtype is also sensitive to CDK4/6 inhibition and enriched in mutations of the PI3K pathway, and targeting both pathways in combination with anti-androgens may be more fruitful [31].

## 4. De-Escalation Opportunities in Early-Stage TNBC

With earlier diagnosis of breast cancer and technological advancement, we have been able to profoundly de-escalate morbid surgical procedures. In addition to de-escalating surgery, further de-escalation could be attained by de-escalating systemic treatment and/or duration in the neoadjuvant or adjuvant settings or using less toxic treatments, such as targeted therapies.

### 4.1. De-Escalation of Systemic Therapy in Early TNBC

Neoadjuvant or adjuvant chemotherapy decreases the risk of recurrence and improves the overall survival, but the absolute benefit in patients with lower recurrence rates would be small, with a potential for long term side effects. Therefore, often patients end up receiving much more systemic treatment than required with fewer benefits and more toxicities. An approach towards personalized treatment considering age, comorbidities, and clinical and biomarker status is needed for addressing the needs of a specific patient.

#### 4.1.1. De-Escalation of Systemic Therapy in Low Clinical Risk Group

Neoadjuvant or adjuvant treatment regimens have not shown absolute benefit in low risk TNBC, which includes small node-negative tumors. Even though we have guidelines for the treatment of breast cancer, there are disparities in the systemic therapy recommendations for early TNBC between these guidelines. TNBC with a tumor size less than 1 cm and without lymph node involvement have a good prognosis, and the omission of chemotherapy can be considered in this group. There are no randomized clinical trials that compare adjuvant chemotherapy vs. no adjuvant chemotherapy in TNBC patients with low risk of recurrence, which includes node-negative small tumors (pT1aN0, pT1bN0). But there were multiple retrospective and observational studies that support these findings. According to a prospective cohort study within the NCCN database, women with pT1a and pT1b TNBC have excellent prognosis without chemotherapy. In pT1aN0 TNBC patients, the 5-year distant relapse-free survival (DRFS) was 93% (95% CI, 84–97%) in the untreated cohort of 74 patients when compared to 100% DRFS in the adjuvant chemotherapy cohort of 25 patients. In the pT1bN0 TNBC patients, the 5-year DRFS was 90% (95% CI, 81–95%) in the cohort of 94 untreated patients and 96% (95% CI, 90–98%) in the cohort of 170 patients who received adjuvant chemotherapy [32]. The 5-year risk of distant relapse was similar in patients who received adjuvant chemotherapy and in those who did not receive adjuvant chemotherapy in both pT1aN0 and pT1bN0 TNBC [7,32].

In a retrospective study of 354 patients with T1N0 TNBC by Ren et al., a significant recurrence-free survival (RFS) benefit was observed in T1c patients (HR = 0.24, 95% CI, 0.08–0.76 with *p* = 0.014), but not in T1b patients with adjuvant chemotherapy (HR = 0.32, 95% CI, 0.03–3.18 with *p* = 0.330) [33]. In another study in pT1N0 patients, adjuvant chemotherapy benefits were mainly observed in pT1c patients rather than pT1a and pT1b patients. In patients with pT1c TNBC, the 5-year RFS rates with and without chemotherapy were 92.8% and 47.2%, respectively (HR = 0.107, 95% CI, 0.047–0.244 with *p* < 0.001). In patients with pT1a TNBC, the 5-year RFS rates with and without chemotherapy were 92.3% vs. 100% (HR = 3.99, 95% CI, 0.005–317.5 with *p* = 0.535) and in pT1b patients, the 5-year RFS with and without chemotherapy were 91.4% vs. 90% (HR = 0.64, 95% CI, 0.05–7.74 with *p* = 0.724) [34].

Another large retrospective study of 4366 patients with early TNBC by Steenbruggen et al. from the Netherlands Cancer Registry showed that adjuvant chemotherapy is associated with better breast cancer specific survival (BCSS) and overall survival (OS) in pT1c tumors but may not outweigh harms in patients with pT1a and pT1b tumors. In the study, BCSS differed with respect to the pathologic tumor sizes (pT1a adjusted HR (aHR) = 4.28, 95% CI, 1.12–16.44; pT1b aHR = 1.12, 95% CI, 0.51–2.49; pT1c aHR = 0.60, 95% CI, 0.43–0.82). The interaction between tumor size and chemotherapy was statistically significant, with *p* value = 0.02. The OS and association between adjuvant chemotherapy was also similar to BCSS (pT1a aHR = 3.52, 95% CI, 1.02–12.14, pT1b aHR = 0.90, 95% CI = 0.48–1.66, pT1c aHR = 0.55, 95% CI = 0.43–0.69). The association between adjuvant chemotherapy and OS was significant (*p* < 0.001) [14].

In another study by de Nonneville et al., the subgroup analysis reported that there was no statistically significant better disease-free and metastasis-free survival in patients with T1a and T1bN0 TNBC who received adjuvant chemotherapy [35]. A recent SEER based study that included 1849 patients demonstrated that T1bN0 TNBC patients had no improvement in BCSS from adjuvant chemotherapy. BCSS was similar in those who received chemotherapy and those who did not receive chemotherapy [36]. De-escalation of adjuvant chemotherapy could be considered for pT1b TNBC patients based on the above retrospective real-world data. If treatment is considered, an anthracycline-sparing approach should be used for pT1b TNBC as discussed below.

#### 4.1.2. De-Escalation by Using Anthracycline-Sparing Approach in Low Risk Early TNBC

The anthracycline and taxane-based chemotherapy backbone is the SOC for neoadjuvant chemotherapy along with pembrolizumab in high-risk early TNBC. However, in stage I tumors (tumor size < 2 cm), anthracycline and taxane-based chemotherapy is still considered the standard of care. We tend to use a dose-dense AC-T regimen in most cases, even if the patient has low-risk disease due to the fear of high recurrence rates. Anthracyclines have shown long-term cardiac side effects, including congestive heart failure (CHF) and cardiomyopathy [16]. Prior studies have observed that the incidence of symptomatic CHF is 5% to 48%, depending on the cumulative doses of anthracyclines [37]. Anthracyclines are well known to cause secondary leukemia, including acute myeloid leukemia (AML) and myelodysplastic syndrome (MDS). According to a database study, the eight-year cumulative incidence of AML and MDS in patients who receive anthracycline-based therapy was 0.90% and 2.24%, respectively. Elderly patients had more risk of developing AML/MDS (incidence was 1.8%) [38]. Therefore, anthracycline-sparing chemotherapy could be considered as an option in elderly patients and those who have high toxicity risks. Hence, the concept of de-escalation of anthracycline-containing regimens has gained more popularity. 

The Anthracyclines in Early Breast Cancer (ABC) trial was one among many trials designed to compare AC-T to TC (taxane-cyclophosphamide) chemotherapy in the adjuvant setting in patients with early-stage HER2 negative breast cancer. TC was found to be slightly inferior to the ACT regimen with regards to the overall four-year invasive disease-free survival (DFS was 88.2% in TC vs. 90.7% in the ACT group with HR of 1.23, 95% CI 1.01–1.50), but anthracycline-based therapy did not improve outcomes in lower risk groups. In the subset analysis of early TNBC, the absolute benefit was more pronounced in the high-risk group that includes lymph node involvement [17]. According to the West German Study Group PlanB trial, six cycles of TC was non-inferior to four cycles of epirubicin/cyclophosphamide, followed by four cycles of docetaxel (EC-T) in patients with HER2 negative early breast cancer with pN0 high genomic risk that included hormone receptor-negative patients or pN1 with genomic intermediate to high-risk disease. In the 5-year outcomes, DFS and OS were similar in the TC and EC-T groups. Similar findings were also observed in the early TNBC subset (*N* = 400) [39]. These trials show that the AC-T regimen may be superior to TC in high-risk disease; however, comparable outcomes have been observed in the lower-risk disease. If considering chemotherapy for pT1b tumors, non-anthracycline-based regimens can be considered as an appropriate alternative for patients with lower-risk early TNBC (e.g., node-negative, <1 cm, or those with cardiac risk factors) and those who prefer to avoid the risks associated with anthracyclines.

Pathologic lymph node staging can be used to guide adjuvant treatment in early TNBC. A retrospective study of 381 patients with early TNBC compared the DFS and OS in patients who received adjuvant taxane-based three-drug chemotherapy (AC-T) and two-drug chemotherapy (TC). They reported that taxane-based triplet adjuvant chemotherapy is superior to doublet in patients with one to nine positive LNs but not in patients with node-negative early TNBC [40]. This further supports de-escalation using anthracycline-sparing therapy for patients with node-negative early TNBC.

Carboplatin-based regimens are a new focus in the development of de-escalation strategies. Many trials incorporating carboplatin with other chemotherapy including taxane-based regimens, PARPi, and immunotherapy are extensively being studied in the neoadjuvant settings, as discussed below.

#### 4.1.3. De-Escalation of Neoadjuvant Systemic Therapy

Carboplatin-based regimens have been investigated as a de-escalating strategy in neoadjuvant treatment of early TNBC. Trials have demonstrated a promising pCR using an anthracycline-free regimen of carboplatin + taxane. In the WSG-ADAPT-TN trial (*N* = 154), patients with Stage I–III TNBC received carboplatin with nab-paclitaxel for four three-week cycles. Pathologic complete response was noted to be 45.9%, which is comparable to the more toxic anthracycline-containing regimens and may be an early predictor of who could be de-escalated, although longer follow up is required to assess the survival data [41]. On further analysis of this trial, pCR was noted to be associated with a basal-like transcriptomic profile, high ki-67, high Prediction Analysis of Microarray 50 (PAM-50) and risk of recurrence score (ROR), and expression of PDL1 and CD8 [42].

In the phase II NEOSTOP trial, Sharma et al. reported similar pCR (54%), EFS, and OS in Stage I–III TNBC patients, with carboplatin + docetaxel (CbD) administered for six three-week cycles compared with carboplatin + standard anthracycline triplet, with overall less toxicity [43]. This pCR rate is comparable to the control arm in the KEYNOTE-522 trial that investigated a combination of anthracycline/cyclophosphamide with carboplatin/paclitaxel [2]. Thus, CbD may serve as a good neoadjuvant anthracycline-free backbone for further studies combining chemotherapy and immunotherapy [43].

The NeoTRIP study (NCT002620280) used a carboplatin-based backbone. This was a phase III randomized controlled trial in which 280 patients with early-stage TNBC were randomized to carboplatin + nab-paclitaxel, with or without immunotherapy, with atezolizumab given every 3 weeks for 8 cycles. Fifty-six percent of patients were PD-L1 positive. The pCR rate was not significantly different between the two study arms (43.5% vs. 40.8%). For patients with PDL1 >5%, pCR was 87.0% vs. 72.0%, PDL1 between 1–5% pCR was 56.2% vs. 44.0%, and PDL1 <1% pCR was 35.1% vs. 41.1% in the treatment arm vs. control arm, respectively. The EFS and OS rates from this study are awaited [44].

These trials indicate that a promising pCR can be achieved using an anthracycline-free regimen with carboplatin and taxanes. Combined with ICIs, we may be able to de-escalate the more toxic chemotherapeutic regimens in the future in a subset of patients. These limited results support further research using anthracycline-free regimens combined with ICIs. The results of the phase II NeoPACT trial, which combines platinum + taxane with pembrolizumab every 3 weeks for 6 cycles, is much awaited (NCT03639948). Table 2 shows the ongoing and completed clinical trials in early TNBC.

#### 4.1.4. De-Escalation of Adjuvant Systemic Therapy

In the GeparNUEVO trial, durvalumab was given only as neoadjuvant along with the standard anthracycline and taxane based chemotherapy; it was not continued in the adjuvant setting for those who attained pCR, yet the study showed significant improvement in the OS and distant DFS (DDFS) [24]. However, currently, the standard of care treatment in the adjuvant setting for patients who successfully achieve pCR after neoadjuvant chemotherapy/pembrolizumab is continued pembrolizumab for 9 cycles based on KEYNOTE-522 findings, with an unclear benefit of pembrolizumab in the adjuvant setting in these patients. Similarly, questions remains as to whether patients who do not attain pCR need both pembrolizumab and capecitabine if BRCA wild type, or a combination of pembrolizumab and olaparib if germline BRCA positive. Future trials should evaluate the role of continuing pembrolizumab in the adjuvant setting for residual disease post neoadjuvant treatment.

With the fear of the aggressiveness of TNBC, most of the current treatments aim to escalate the available treatment regimens. The above data show that there is a huge potential for de-escalating treatment options in early TNBC, both in the adjuvant and in the neoadjuvant settings without compromising the effectiveness of the treatments. Systemic chemotherapy can be omitted in very low-risk early TNBC patients, and de-escalation can also be executed by carefully selecting patients who need systemic treatment through individual risk assessment. Carboplatin-based treatment regimens can be considered as a new potential anthracycline-sparing de-escalation strategy.

## 5. Biomarkers and Imaging to Guide Systemic Therapy De-Escalation

We summarize the data regarding recent advances in novel biomarkers, knowledge of mutations for targeted therapies, and the use of early imaging modalities to guide personalized decision making. The use of these strategies may potentially pave the way for de-escalating aggressive regimens.

### 5.1. Prognostic and Predictive Biomarkers

#### 5.1.1. Tumor Microenvironment Biomarkers Predicting pCR

Several studies have shown that stromal tumor infiltrating lymphocytes (sTILs) play an important role in prognosis and response to chemotherapy in patients with TNBC [45]. In 2010, Denkert et al. reported that high sTILs in breast cancer are a predictor of pCR to NACT with pCR rates of 40–42% in the cohort with high sTILs vs. 3–7% in the cohort with low TILs [46]. TILs were then examined in an analysis of two randomized phase III adjuvant French studies in TNBC patients, which showed that high sTILs correlated with better ten-year OS (89% vs. 68%). However, they were not found to be predictive for response to anthracycline-based chemotherapy [47]. In a retrospective study from the Netherlands in 481 young (<40 years old) early-stage TNBC patients who only underwent surgery, De Jong et al. found that TIL expression levels correlate with overall survival (OS) and distant recurrence-free survival (DRFS). They found that the OS at 15 years for TIL <30%, 30–75%, and >75% was 59%, 76%, and 93%, respectively, and DRFS at 15 years was 67%, 83%, and 98%, respectively [48]. In a pooled analysis from four TNBC cohorts of early-stage mostly node-negative (83%) TNBC patients, Park et al. demonstrated excellent survival outcomes without systemic therapy in patients with sTIL >30%. The 3-year invasive disease-free survival (iDFS) was 93%, DDF 97%, and OS 99% [49]. These survival outcomes are similar to a pooled analysis from nine studies by Loi et al. in which a similar group of node negative early TNBC patients who received anthracycline-based chemotherapy with sTILs >30% had a 3-year iDFS 92%, DDFS 97%, and OS 92% [50]. These studies show that sTILs may be able to identify a subgroup of patients with Stage 1 TNBC with an excellent prognosis, in which systemic therapy may be able to be de-escalated or omitted altogether. A recent large meta-analysis shows TILs to be both prognostic for favorable long-term clinical outcomes as well as predictive for pCR among TNBC [51]. However, this evidence is mostly from retrospective studies and needs confirmation in prospective cohorts. Recent studies suggest that sTILs could possibly be added in the 8th edition of the American Joint Committee on Cancer (AJCC) staging system to up- or downstage early TNBC [52]. 

Similarly, single cell spatial analysis from the NeoTRIPaPDL1 trial (NCT02620280) found that GATA3 and CD20 in the tumor microenvironment, HLA-DR on the epithelial cells, and Ki67 both on the tumor microenvironment and the epithelial cells, were significant for their predictive ability for atezolizumab benefit. Expression of these biomarkers above the median was linked to a pCR rate increase of 10% or more (*p* < 0.05). It was also noted that higher expression of two cell phenotypes, PD-L1 positive, IDO-positive antigen presenting cells (APCs) and CD56-positive neuroendocrine (NE) epithelial cell, was associated with a higher pCR when treated with atezolizumab. In patients with PD-L1 positive, IDO-positive APCs who received atezolizumab, pCR was 64.6% vs. 24.6% for those with high and low expression, respectively (*p* < 0.001) [53].

#### 5.1.2. PD-L1 as a Predictor of pCR

Immune checkpoint inhibitors targeting PD-1 or PD-L1 have become the standard of care in many solid tumor types. In TNBC, PD-L1 expression has been estimated to be 40–65% on the immune cells [54]. Nineteen percent of tumor cells were PD-L1 positive, defined by >5% membranous staining by IHC [55]. PD-L1 expression was investigated as a biomarker of response to these therapies; however, even patients who are PD-L1 negative respond to these agents. Therefore, there is a lack of a quantitative association between PD-L1 expression and response.

#### 5.1.3. Immune Gene Signature as a Predictor of pCR

Multi-gene signature has been studied as a comprehensive tool that can capture the immunogenicity of TNBC. The GeparSixto trial was analyzed for mRNA markers and showed that an immune signature composed of seven immune-activating genes (CXCL9, CCL5, CD8A, CD80, CXCL13, IGKC, CD21) and five immunosuppressive genes (IDO1, PD-1, PD-L1, CTLA4, FOXP3) was validated as a marker for immune reaction. The increased mRNA expression level of these genes, including immunosuppressive genes, was associated with pCR [56].

#### 5.1.4. Circulating Tumor DNA (ctDNA) as a Predictor of pCR

Circulating tumor DNA (ctDNA) is the fragmented DNA released into the bloodstream from the necrosis of the tumor tissue. The detection of ctDNA has been progressively used in studies to demonstrate its predictive role in identifying minimal residual disease after neoadjuvant chemotherapy in early TNBC and thereby identifying the high-risk patients for recurrence. Riva et al., in a prospective study, demonstrated that ctDNA levels are associated with tumor proliferation rate and can be used to monitor tumor progression during NACT. They also found that those who had a slow decrease of ctDNA level during NACT had shorter survival [57]. In the BRE12-158 clinical trial that enrolled early-stage TNBC patients who had residual disease after the NACT, ctDNA was positive in 63% of patients (90 out of 142). The secondary analysis of the trial showed that detection of ctDNA and circulating tumor cells (CTCs) after NACT in patients with early-stage TNBC is significantly associated with inferior DDFS, DFS, and OS [58]. Similar results were seen in another study, which showed that next generation ctDNA sequencing of patients with early TNBC who did not attain pCR after NACT could predict recurrence with high specificity, and they had inferior DFS (median DFS: 4.6 months vs. not reached; HR = 12.6, 95% CI: 3.06–52.2, *p* < 0.0001). However, the sensitivity of detection of ctDNA was low in the study as they could identify the ctDNA in the plasma sample of only 4 out of 33 patients who had somatic mutations [59]. In a study by Magbanua et al., the authors found that high-risk early breast cancer patients who did not clear ctDNA during the NACT were more likely to have residual disease than those who cleared the ctDNA. The ctDNA was detected in 73% of patients (61 out of 84) pretreatment. The ctDNA positivity decreased during the NACT, and only 8.6% (*N* = 5) of patients remained ctDNA positive after completion of the NACT. In this study, all patients who attained pCR were ctDNA negative. An important finding was that patients who did not achieve pCR but were ctDNA negative had improved survival, comparable to those who attained pCR (HR 1.4, 95% CI 0.15–13.5) [60]. With more advancements in studies, ctDNA can be used as a reliable biomarker to identify a subgroup of patients who have a comparatively lower chance of disease recurrence after NACT in whom the adjuvant treatments could be effectively avoided.

### 5.2. Targeted Strategies to Improve pCR

#### 5.2.1. Tumor-Associated Macrophages in the Tumor Microenvironment

Therapies targeting the tumor microenvironment (TME) are also currently under investigation. Tumor-associated macrophages (TAMs) are known to promote the progression and metastasis of TNBC by releasing inhibitory cytokines, reducing the functions of TILs, promoting regulatory T-cells (Tregs), and modulating the expression of PD-1/PD-L1 on the TME [61]. Cabiralizumab is a monoclonal antibody that blocks colony stimulating factor-1 receptor (CSF1R) and has demonstrated the ability to block activation of monocytes and macrophages. The combination of this antibody with immunotherapy (nivolumab) and an anthracycline-free chemotherapy regimen (carboplatin + paclitaxel) is being studied in the neoadjuvant setting, with the central hypothesis that it would decrease tumor-associated macrophages (TAMs) and increase TILs, thereby improving outcomes. The primary outcome measure of this study includes the percentage change in TILs and TAMs, with pCR and RFS being looked at as secondary outcome measures (NCT04331067). Such novel agents, if found to be efficacious, may provide an alternative to current standard of care systemic therapy, thereby helping to minimize treatment related toxicity while maintaining excellent efficacy.

#### 5.2.2. PARP Inhibitors for Germline BRCA Mutation

Among the patients with TNBC, approximately 10–30% have germline BRCA (gBRCA) mutations. Approximately 80% of breast cancers that occur in patients with gBRCA1 mutations are triple-negative with a basal-like profile. BRCA 1 and BRCA 2 are tumor suppressor genes that belong to the homologous recombination (HR) repair pathway that repairs double-strand DNA breaks. Platinum-based regimens are a focus of interest in several trials in patients with BRCA mutations. Cisplatin every three weeks for 4 cycles was evaluated in a randomized phase II INFORM clinical trial of neoadjuvant cisplatin vs. AC in gBRCA carriers (70% TNBC patients). The pCR rate was 18% with cisplatin and 26% with AC [62]. Poly ADP-ribose polymerase inhibitors (PARPi) showed efficacy in patients with BRCA mutations. Poly ADP-ribose polymerase (PARP) 1 is a protein that facilitates the DNA repair process. PARPi traps PARP1 and induces cell death by preventing single-stranded break repair, followed by double-stranded breaks without functional homologous recombination in patients with BRCA mutations [6]. Talazoparib has been approved for patients with locally advanced or metastatic, HER2-negative breast cancer with deleterious gBRCA mutations [63]. 

There are several ongoing studies to evaluate the role of PARPi as a neoadjuvant treatment in early-stage BRCA mutated breast cancer. MD Anderson reported a study of neoadjuvant talazoparib in patients with gBRCA mutations (NCT02282345). TNBC patients consisted of 15 out of the 20 patients enrolled, and 53% achieved pCR after six months of single agent talazoparib. In this trial, patients subsequently received adjuvant standard chemotherapy based on physician’s discretion [64]. These results supported the larger neoadjuvant phase II nonrandomized NEOTALA study, which investigated single agent talazoparib in gBRCA 1/2 mutated early HER2 negative breast cancer (NCT03499353). This study included patients with early TNBC, and they received 24 weeks of neoadjuvant talazoparib and then underwent surgery. Neoadjuvant talazoparib monotherapy resulted in pCR in 45.8% of evaluable patients (48 patients) and 49.2% in the intent to treat population (61 patients). This was comparable to standard combination anthracycline and taxane regimen, and the treatment was tolerated well [65]. This regimen could be especially useful for select patients where chemotherapy is contraindicated; for example, those exposed to prior chemotherapy for other cancers or those with a poor performance status where the treating clinician may not want to consider giving intensive chemotherapy/pembrolizumab in the neoadjuvant setting.

#### 5.2.3. PI3K/AKT/mTOR Targeted Therapies

Mutations in PIK3CA, AKT, PTEN, or mTOR can activate the Phosphatidylinositol-3-kinase (PI3K) pathway, leading to cell growth. Deregulation of any PI3K pathway component has been seen in up to 50% of patients and are seen amongst all molecular subtypes. Ipatasertib, a highly selective pan-AKT small molecule inhibitor, was studied initially in the phase II LOTUS trial in the metastatic setting and showed a significant PFS improvement in patients with alteration in the PIK3CA/AKT/PTEN pathway [66]. Subsequently, it was studied in neoadjuvant early TNBC in the phase II FAIRLANE study. Weekly paclitaxel × 12 weeks plus ipatasertib or placebo (days 1–21 every 28 days) was given to a patient population that contained both low-PTEN and PTEN-altered tumors. There was an increase in pCR from 13% to 17% in the ipatasertib arm in the ITT population (*N* = 151, 95% CI −9.0 to 16.5), which was not statistically significant. In patients with low PTEN, pCR was 16% vs. 13% in placebo, and in patients with altered PTEN, pCR was 18% vs. 12% in placebo, which was not statistically significant. The addition of ipatasertib did not significantly increase pCR rates. The overall pCR rates in this study are much less than typically expected in early TNBC, likely due to the short duration of treatment of 12 weeks and utilization of only paclitaxel as chemotherapy. In addition, there was no significant difference in pCR in PTEN mutated patients who received ipatasertib compared with those who did not have the mutation. This is likely due to the significant heterogeneity among TNBC. Though this was a negative trial, these results support further evaluation of this pathway in combination with chemotherapy [67].

#### 5.2.4. Epidermal Growth Factor Receptor (EGFR) Targeted Therapies

EGFR overexpression can be used as a target in TNBC as 60% of triple negative tumors have EGFR expression. EGFR expression has been recognized as a poor prognostic factor in TNBC [68]. EGFR inhibitors, including Tyrosine kinase inhibitors (TKI) and monoclonal antibodies (mABs), have been used in multiple early phase clinical trials in the past, but the results have been mostly disappointing [8,69]. A neoadjuvant study using EGFR inhibitor cetuximab in combination with ixabepilone (NCT01097642) has recently completed accrual, and the final analysis is awaited. Inhibition of EGFR may be another targetable pathway that could be used to de-escalate treatment if EGFR inhibitors show benefit in ongoing clinical trials.

#### 5.2.5. Antiangiogenic Agents

Vascular Endothelial Growth Factor (VEGF) inhibitors impair the neovasculature of the tumor, thus impairing tumor growth. Bevacizumab, an anti-angiogenic monoclonal antibody against VEGFR, has been evaluated in multiple studies in TNBC, especially in the metastatic setting. Most of the studies tend to escalate the treatment by the addition of bevacizumab to standard NACT regimens [6]. The addition of bevacizumab to anthracycline and taxane-based adjuvant chemotherapy in the BEATRICE study did not show a statistically significant improvement in the DFS or OS [70]. The use of bevacizumab in the neoadjuvant setting in stage II/III TNBC with or without carboplatin concurrent with AC-T was studied in the CALGB 40603/Alliance trial. The addition of either carboplatin or bevacizumab to the standard neoadjuvant chemotherapy increased the pCR rates but did not show improvement in long term outcomes [71,72]. VEGF inhibition continues to remain a potential pathway that can be used for developing targeted treatments in the (neo) adjuvant setting in early TNBC.

#### 5.2.6. Androgen Receptor Targeting

Almost 10–40% of TNBC expresses androgen receptors (AR); this makes AR a potential target for treatment. Androgen Receptor inhibitors abiraterone and enzalutamide have shown clinical benefit in AR positive (>/=10% by Immunohistochemistry) metastatic TNBC [73,74]. This has led us to study the benefits of AR-targeted therapy in the neoadjuvant setting in early TNBC. A phase II trial studying the efficacy of enzalutamide and paclitaxel in the neoadjuvant setting in patients with Stage I–III AR-positive TNBC is actively accruing (NCT02689427). If the study shows promising results, enzalutamide with minimal chemotherapy can be used to de-escalate the current complex neoadjuvant regimen in early TNBC.

### 5.3. Novel Clinical Trial Design Based on Imaging Response

Trials have attempted to tailor therapy by identifying subsets of chemo-sensitive and chemo-resistant breast cancer. In the ARTEMIS trial, response was assessed in patients with early TNBC after four cycles of NACT with doxorubicin + cyclophosphamide (AC). Those who had more than 70% volumetric reduction, i.e., chemo-sensitive disease, continued with standard of care taxane-based therapy with or without platinum. Patients who had chemo-insensitive disease were given the option to be randomized to either of four Phase II clinical trials based on transcriptomic and genomic profiling results (NCT02276443). These different groups consist of a combination of targeted agents such as androgen-receptor blocker, PDL1-inhibitor, EGFR monoclonal antibody, or VEGF inhibitor with chemotherapy (NCT02689427, NCT02530489, NCT02593175, NCT02456857). Results from this trial and other similar studies may help identify agents that might be effective in TNBC, especially in those with chemo-resistant disease. Thereby, this sets up a foundation to enable us to de-escalate management in selected subgroups of patients with early TNBC and possibly even replace traditional chemotherapy with targeted agents. Knowledge from this trial, guided by imaging response along with the other completed trials exploring checkpoint inhibitors and targeted agents, should be used to design novel clinical trials for treatment de-escalation.

Many biomarkers are currently being studied to help guide decision making regarding systemic therapy in early TNBC by their predictive and/or prognostic value. The presence of sTILs and the absence of ctDNA after NACT is associated with better outcomes and may help identify cohorts of patients who may be spared adjuvant treatment. PD-L1 is another biomarker but is not very sensitive and/or specific and thus we continue to use PD-1/PD-L1 therapies in both those who are positive and negative. Targeted treatments such as single agent PARPi in BRCA mutated patients in the neoadjuvant setting have shown results comparable to conventional chemotherapy and may be a chemotherapy-sparing option in suitable patients. Other biomarkers, such as immune gene signatures and other targeted treatments, including inhibitors of TAMs, PI3K/AKT/mTOR, EGFR, VEGF, and AR, are currently being studied in this patient population, and we await results that may further enable us to offer personalized options, resulting in de-escalation of treatment.

## 6. Conclusions and Future Direction

Triple-negative breast cancer is a heterogenous, aggressive breast cancer that has high recurrence rates. Anthracycline and taxane-based chemotherapy with neoadjuvant pembrolizumab has become the standard of care for patients with TNBC with T1c, nodal stage N1-2, or tumor stage T2-4, nodal stage N0-2. The literature shows that pT1b tumors could potentially be spared chemotherapy, and pT1c tumors could derive significant benefit from taxane-based chemotherapy, thus sparing the use of anthracyclines. Treatments targeting mutations and making use of tissue biomarkers or early response on imaging in TNBC are appealing strategies to tailor treatment to a particular tumor, and hence, de-escalate treatment. Better risk stratifications based on molecular profiling of the tumor and the use of predictive biomarkers may minimize the need for systemic therapies. Current trials ongoing in the field of targeted therapies in TNBC include the use of PI3K/AKT/mTOR inhibitors, EGFR targeted agents, androgen receptor inhibitors, and antiangiogenic agents that may lead to the discovery of novel avenues to de-escalate treatment. Future prospective trials should also evaluate incorporating ctDNA after NACT into decision making for adjuvant therapy. As we understand more of the biology and heterogeneity of TNBC, we also expect further targets to be elucidated and therapies to be tested, which will further enable de-escalation. We are currently investigating the impact of short duration immunomodulating therapies to improve the tumor microenvironment (chemokine modulation consisting of interferon-alpha, TLR3 agonist, and COX-2 inhibitor), given along with standard chemotherapy in the neoadjuvant setting in early TNBC in a phase 1 clinical trial (NCT04081389), which may help us avoid giving immune therapies for an extended duration, especially after surgery in the adjuvant setting [75,76,77]. In the adjuvant setting after completion of neoadjuvant therapy, the OptimICE-pCR clinical trial will study the clinical outcomes of adjuvant pembrolizumab vs. observation in early TNBC patients who received NACT with pembrolizumab. This would be another de-escalation opportunity if the results are promising. The results of the other ongoing clinical trials with the addition of immunotherapy in patients who have residual disease after neoadjuvant chemotherapy—the A-BRAVE trial (adjuvant anti-PDL1 antibody avelumab), NCT02926196 and the SWOG1418/BR006 trial (adjuvant pembrolizumab), NCT02954874—would help us to determine if immunotherapy given in the adjuvant setting alone would be sufficient in this patient population. This could be a major de-escalation strategy in early TNBC patients if the results are encouraging.

There is an urgent need to develop personalized treatments tailored to patients based on their clinical characteristics, comorbidities, and biomarker status, which would provide maximum benefit while curtailing side effects.

## Figures and Tables

**Table 1:** Adjuvant/Neoadjuvant chemotherapy recommendations for stage I TNBC according to various international guideline.

Stage	AJCC Stage Definition	International Guideline	Recommendation
T1aN0M0	Tumor >1 mm but ≤ 5 mm in greatest dimension, no evidence of regional LN metastasis identified	NCCN [10]	No adjuvant therapy (category 2A). Adjuvant chemotherapy may be considered in patients with high-risk features (e.g., young patients with high grade histology) (category 2B)
		ASCO [11]	Should not routinely offer Neoadjuvant therapy
		St.Gallen [13]	No adjuvant chemotherapy
		Dutch [14,15]	No adjuvant chemotherapy
T1bN0M0	Tumor > 5 mm but </=10 mm in greatest dimension, no evidence of regional LN metastasis identified	NCCN [10]	Consider adjuvant chemotherapy (category 2A)
		ASCO [11]	Should not routinely offer neoadjuvant therapy
		St.Gallen [13]	Adjuvant chemotherapy
		Dutch [14,15]	No adjuvant chemotherapy
T1cN0M0	Tumor > 10 mm but </=20 mm in greatest dimension, no evidence of regional LN metastasis identified	NCCN [10]	Adjuvant chemotherapy (category 1)
		ASCO [11]	Offer neoadjuvant therapy
		St.Gallen [13]	Adjuvant chemotherapy
		Dutch [14,15]	Adjuvant chemotherapy recommended if tumor grade 3 or if >/=grade 2 and age </= 35 years

**Table 2:** Summary of the ongoing and completed clinical trials that provide an opportunity to de-escalate the current standard neoadjuvant regimen for early TNBC patients.

ONGOING CLINICAL TRIALS										
Trial Name/Identifier	Variable	Study Type, Phase, Estimated Enrollment	Study Design	Population	Setting	Intervention	Primary Outcome Measures	Secondary Outcome Measures	Estimated Primary Completion Date	Results
NeoTRIPaPDL1/NCT02620280	PDL1	Interventional, Phase 3, *N* = 278	RCT	early-stage TNBC	Neoadjuvant	Carboplatin (AUC2 IV on day 1 and day 8), Abraxane (125 mg/m^2^ IV on day 1 and day 8), +/−Atezolizumab (1200 mg IV on day 1) for 8 cycles, surgery, followed by AC/EC/or FEC for 4 cycles	EFS	pCR, DFS, Adverse events	May 2022	interim results: pCR data resulted 43.5% with atezolizumab vs. 40.8% without OR 1.11
NeoPACT/NCT03639948	PDL1	Interventional, Phase 2, *N* = 100	single arm	early-stage TNBC	Neoadjuvant	Carboplatin (AUC: 6 IV), Docetaxel (75 mg/m^2^, IV), Pembrolizumab (200 mg, IV) every 21 days for 6 cycles	pCR	MRD, RFS	November 2024	
NeoSTAR/NCT04230109	Antibody-Drug Conjugate, PDL1	Interventional, Phase 2, *N* = 51	two separate cohorts	early-stage TNBC	Neoadjuvant	Monotherapy cohort: Sacituzumab Govitecan IV (D1 and 8 per 21-day cycle) for 4 cycles (monotherapy cohort), Combination Cohort: Sacituzumab Govitecan IV + Pembrolizumab IV (per 21-day cycle), for 4 cycles; if complete response may proceed directly to surgery, if not chemotherapy per physician	pCR at 12 weeks	DFS, OS, BCS rate, Adverse Events, QoL	October 2024	
IMpassion031 NCT03197935	PDL1	Interventional, Phase 3, *N* = 333	RCT	early-stage TNBC	Neoadjuvant	Atezolizumab (840 mg) IV q2 weeks with nab-paclitaxel (125 mg/m^2^) Q1 week for 12 weeks, followed by atezolizumab (840 mg) q2 weeks with doxorubicin (60 mg/m^2^) and cyclophosphamide (600 mg/m^2^) q2 weeks for 4 doses. Followed by adjuvant atezolizumab at a fixed dose of 1200 mg IV q3 weeks for 11 doses, for a total of approximately 12 months of atezolizumab therapy.	pCR in ITT, pCR in PDL1 positive group	EFS, DFS, OS, Adverse Events,	October 2022	Improved pCR in atezolizumab group (57.6% vs. 41%), and in PDl1 positive group (68.8% vs. 49.3%.
NCT04331067	TIL	Interventional, Phase 1b/2, *N* = 50	RCT	Stage II/III TNBC	Neoadjuvant	Paclitaxel 80 mg/m^2^ weekly, Carboplatin AUC 5 q3 weeks, Nivolumab 240 mg q2 weeks all for 12 weeks +/− Cabiralizumab 4 mg/kg q2 weeks for 2 weeks	% change in TILs and TAMs, safety	pCR, RFS, safety	December 2022	
NCT02689427	ARi with paclitaxel	Interventional, Phase IIB, *N* = 37	single arm	Stage I–III AR positive TNBC	Neoadjuvant	enzalutamide PO QD on days 1–7 and paclitaxel IV on D1, repeated every 7 days for upto 12 cycles followed by surgery	pCR and residual cancer burden index,	PFS, biomarker response level	June 2023	
**COMPLETED CLINICAL TRIALS**										
**Trial Name/Identifier**	**Variable**	**Study Type, Phase, Estimated Enrollment**	**Study Design**	**Population**	**Setting**	**Intervention**	**Primary Outcome Measures**	**Secondary Outcome Measures**	**Estimated Primary Completion Date**	**Results**
GeparNUEVO NCT02685059	PDL1	Interventional, Phase 2, *N* = 174	RCT	early-stage TNBC	Neoadjuvant	Durvalumab/placebo monotherapy (0.75 g i.v.) for the first 2 weeks (window phase), followed by D/placebo plus nab-paclitaxel 125 mg/m² weekly for 12 weeks, followed by D/placebo plus epirubicin/cyclophosphamide (EC) q2 weeks for 4 cycles.	pCR	pCR per arm, clinical response, BCR, Molecular markers, gene expression, toxicity and compliance, survival	March 2018	Improved long term outcomes in durvalumab group
NeoTALA NCT03499353	PARPi	Interventional, Phase 2, *N* = 61	single arm	early-stage HER2 negative breast cancer with BRCA 1 or 2 mutation	Neoadjuvant	Talazoparib PO 1 mg per day for 24 weeks followed by surgery	pCR in evaluable analysis set	pCR in ITT analysis set, residual cancer burden, probability of being event free at 3 years, probability of being alive at 3 years, AE	September 2020	interim results: pCR in 45.8% in evaluable and 49.2% in intention to treat patients, terminated due to change in clinical development strategy
WSG-ADAPT-TN trial, NCT01815242		Interventional, Phase 2, *N* = 336	RCT	early-stage TNBC	Neoadjuvant	nab-paclitaxel 125 mg/m^2^/gemcitabine 1000 mg/m^2^ d1,8 q3w vs.nab-paclitaxel 125 mg/m^2^/carboplatin AUC2 day 1,8 q3w for 4 cycles	pCR	None	May 2020	pCR was higher in nab-paclitaxel/carboplatin group (45.9% vs. 28.7%)
NeoSTOP NCT02413320		Interventional, Phase 2, *N* = 101	RCT	early-stage TNBC	Neoadjuvant	Carboplatin + docetaxel q3weeks for 4 cycles comapred with carboplatin + standard ACT	pCR	MRD	February 2020	pCR (54%), EFS, OS similar in both groups
FAIRLANE NCT02301988	PIK3CA/AKT/mTOR	Interventional, Phase 2, *N* = 151	RCT	early-stage TNBC	Neoadjuvant	Ipatasertib vs. placebo orally daily on Days 1–21 of each 28-day cycle for 3 cycles and paclitaxel IV qweek for 3 cycles (12 total doses)	pCR	ORR, AE	August 2017	Increase in pCR in the ipatasertib group, but was not statistically significant (17% vs. 13%)
NCT01097642	EGFR	Interventional, Phase 2, *N* = 40	RCT	early-stage TNBC	Neoadjuvant	Ixabepilone alone vs. Ixabepilone given in combination with cetuximab	pCR	RFS, safety and toxicity	December 2019	Pending results
NCT02282345	PARPi	Interventional, Phase 2, *N* = 33	single arm	early-stage breast cancer w/BRCA 1 or 2 mutations, 15/20 are TNBC	Neoadjuvant	Talazoparib PO QD on days 1–28 for 6 cycles, followed by standard of care per physician	No of patients enrolled, toxicity	Clinical response	April 2021	53% pCR with single agent talazoparib.

Abbreviations: AC = Adriamycin and Cyclophosphamide; Ari = Androgen Receptor Inhibitor, AE = Adverse Effects, AUC= Area Under Curve; ACT = Anthracycline, cyclophosphamide, paclitaxel; BCR = Breast Conservation Rate, CSF1R = Colony Stimulating Factor-1 Receptor, DEFS = Distant Event Free Survival; EC = Epirubicin and Cyclophosphamide; ECOG = Eastern Cooperative Oncology Group; EFS = Event Free Survival; FEC = Fluorouracil, Epirubicin hydrochloride, and Cyclophosphamide; ICR = Independent Central Review; ITT = Intention-to-Treat; MRD = Minimal residual disease; OR = Odds Ratio; ORR = Objective response rate; PARPi = Poly-ADP Ribose Polymerase Inhibitors; pCR = Pathological complete response; PD-L1 = Programmed Death-Ligand 1; PFS = Progression-free survival; RCT = Randomized Controlled Trial; RECIST = Response Evaluation Criteria In Solid Tumors; RFS = Recurrence-free survival; TEAEs = Treatment-Emergent Adverse Events; TNBC = Triple Negative Breast Cancer; VEGF = Vascular Endothelial Growth Factor.
